# Dietary Intakes of Vegetable Protein, Folate, and Vitamins B-6 and B-12 Are Partially Correlated with Physical Functioning of Dutch Older Adults Using Copula Graphical Models

**DOI:** 10.1093/jn/nxz269

**Published:** 2019-12-20

**Authors:** Pariya Behrouzi, Pol Grootswagers, Paul L C Keizer, Ellen T H C Smeets, Edith J M Feskens, Lisette C P G M de Groot, Fred A van Eeuwijk

**Affiliations:** 1 Biometris, Mathematical and Statistical Methods, Wageningen University and Research, Wageningen, Netherlands; 2 Department of Human Nutrition, Wageningen University and Research, Wageningen, Netherlands

**Keywords:** copula graphical models, nutrient networks, muscle health, physical functioning, older adults, sarcopenia

## Abstract

**Background:**

In nutritional epidemiology, dealing with confounding and complex internutrient relations are major challenges. An often-used approach is dietary pattern analyses, such as principal component analysis, to deal with internutrient correlations, and to more closely resemble the true way nutrients are consumed. However, despite these improvements, these approaches still require subjective decisions in the preselection of food groups. Moreover, they do not make efficient use of multivariate dietary data, because they detect only marginal associations. We propose the use of copula graphical models (CGMs) to model and make statistical inferences regarding complex associations among variables in multivariate data, where associations between all variables can be learned simultaneously.

**Objective:**

We aimed to reconstruct nutritional intake and physical functioning networks in Dutch older adults by applying a CGM.

**Methods:**

We addressed this issue by uncovering the pairwise associations between variables while correcting for the effect of remaining variables. More specifically, we used a CGM to infer the precision matrix, which contains all the conditional independence relations between nodes in the graph. The nonzero elements of the precision matrix indicate the presence of a direct association. We applied this method to reconstruct nutrient–physical functioning networks from the combined data of 4 studies (Nu-Age, ProMuscle, ProMO, and V-Fit, total *n* = 662, mean ± SD age = 75 ± 7 y). The method was implemented in the R package *nutriNetwork* which is freely available at https://cran.r-project.org/web/packages/nutriNetwork.

**Results:**

Greater intakes of vegetable protein and vitamin B-6 were partially correlated with higher scores on the total Short Physical Performance Battery (SPPB) and the chair rise test. Greater intakes of vitamin B-12 and folate were partially correlated with higher scores on the chair rise test and the total SPPB, respectively.

**Conclusions:**

We determined that vegetable protein, vitamin B-6, folate, and vitamin B-12 intakes are partially correlated with improved functional outcome measurements in Dutch older adults.

## Introduction

In studying relations between nutritional intake and health-related outcomes, dealing with confounding is a major challenge. A straightforward and frequently used approach is via linear models, which adjusts for important covariates that confound the association. These covariates, however, have to be selected a priori, and this selection is therefore prone to subjectivity. Many other approaches have arisen to (partly) overcome the issues in dealing with confounders. In the last few years, more and more researchers have used dietary pattern analyses, such as principal component analysis and cluster analysis, to deal with internutrient correlations, and to more closely resemble the true way nutrients are consumed. However, despite these improvements, these approaches still require subjective decisions in the preselection of food groups (1). Furthermore, these approaches only inform about relations between dietary patterns and outcomes, which do not allow for mechanistic explanations and for identification of the exact nutritional factors that need improvements (2).

Graphical models are a powerful class of statistical models for reconstructing the complex relations between variables in multivariate data (3). The key feature of graphical models is to uncover *conditional dependence* relations, meaning that 2 variables are connected by an edge if and only if they are dependent after all other variables are accounted for. The detection of conditional (in)dependence relations through graphical models is a key component of the statistical analysis of observational studies. Graphical models, and specifically undirected graphical models, have been used extensively in genetics (4–9), metabolomics (10, 11), and epidemiology (12). There are different types of graphical models [see, e.g., (13)]; one specific type of graphical model is called the copula graphical model (CGM), which can deal with ordinal data, non-Gaussian data, and mixed ordinal/continuous data. A Gaussian CGM is the simplest possible multivariate ordinal (or mixed ordinal/continuous) model because it uses the lowest number [${\rm{O}}( {{\rm{\ }}{p^2}} )$ for $p\ $number of variables or, to be precise, $p\ ( {p - 1} )/2$] of parameters to describe the full multivariate dependence. Other multivariate models for ordinal data (or mixed ordinal/continuous data) usually require estimating larger number of parameters, which make them more complex to be applied to high-dimensional data.

Recently, Iqbal et al. (14) used Gaussian graphical models for identifying conditional independence structures between food intake variables in dietary intake data to understand the eating behavior of German adults. The Gaussian graphical model assumes that data follow a multivariate Gaussian distribution. However, multivariate data sets arising from food and nutrition science typically accommodate different variable types. Thus, in this article we focus on CGMs (15–17) which can be applied to any study that involves a mixture of binary, ordinal, and non-Gaussian variables. Recently, CGMs have been used to detect dietary meal networks (18). Here, we use the methodology developed by Behrouzi and Wit (15) to learn complex association patterns that exist among nutrient intake, physical performance, and muscle strength. This method may help to identify conditional intakes of different nutrients to prevent the progressive loss of muscle mass and muscle strength and ultimately to understand the process involved in aging.

Dietary adjustments might provide feasible strategies to promote healthy ageing. Therefore, understanding the relations between dietary intake and physical functioning in older adults is of great interest. However, decisions regarding new dietary strategies for healthy ageing cannot be based on the result of a single study, because results typically vary from one study to another. Rather, it is necessary to synthesize data across studies. Here we integrated data from multiple studies, where we have combined the most overlapping dietary studies that included dietary intake, physical performance, and muscle strength, carried out within the same center (Wageningen University, Netherlands). Given the aforementioned considerations, we combined the baseline data of 4 studies: Nu-Age (19), ProMuscle (20), ProMO (21), and V-Fit (22).

The key objective of this article is therefore to implement the CGM on the combined data (as well as on each individual study) to reveal conditional dependence relations between muscle function variables (handgrip, different functional tests), nutrients, and other covariates (e.g., age, gender, and BMI). Studying underlying partial correlation networks jointly for dietary intake and nutrient components along with muscle strength and physical performance variables may indicate which nutrients play important roles in age-related functional decline.

## Methods

### Assessment of dietary intake and physical functioning in the 4 substudies

The inclusion and exclusion criteria of the 4 substudies and the dietary assessment methods they used are shown in **Table 1**. All subjects in all studies gave written informed consent and all studies were approved by the Wageningen University Medical Ethical Committee. The substudies that started after 2005 (Nu-Age, ProMuscle, and ProMO) were registered at clinicaltrials.gov.

**TABLE 1:** Characteristics of the 4 included studies^1^

Study name	Nu-Age	ProMuscle	ProMO	V-Fit
Years of enrolment	2012–2014	2009–2010	2016–2017	1997
Inclusion criteria	Aged 65–79 y	>65 y old, frail or prefrail according to Fried criteria	>65 y old, malnutrition (score <12 on MNA-SF)	>70 y old, using care service, BMI ≤25 kg/m^2^ or unintentional weight loss
Exclusion criteria	Frail according to Fried criteria Malnutrition Dementia Major chronic diseases Severe heart disease Insulin-treated diabetes	Participation in resistance-type exercise programs in 2 y before study eGFR <60 mL · min^−1^ · 1.73m^−2^ Any present form of cancer COPD Diabetes	Resistance exercise >2 h/wk Life expectancy <12 mo eGFR <30 mL · min^−1^ · 1.73m^−2^ Use of diabetes medication Use of >21 alcohol units per week	Regular exercise Institutionalized Terminal disease Taking multivitamins
Dietary assessment method	7-d food records	3-d food records	2-d food records	3-d food records

^1^COPD, chronic obstructive pulmonary disease; eGFR, estimated glomerular filtration rate; MNA-SF, Mini Nutritional Assessment Tool—Short Form.

### Nu-Age

The participants of the Nu-Age trial (*n* *=* 252; NCT01754012) were recruited via invitation letters sent to all older adults living in apartment buildings in the surroundings of Wageningen and Arnhem, Netherlands. Participants were independently living nonfrail older adults aged 65–79 y, free of malnutrition, dementia, major chronic diseases, severe heart disease, and type 1 or insulin-treated type 2 diabetes.

Participants filled out food records on 7 consecutive days. Participants were trained in reporting foods, portion sizes, and preparation methods. Trained dietitians or research nutritionists assessed the food records for completeness during a home visit. Nutrient content was calculated by the Dutch food composition database of 2011. Frequency, type, brand name, and dose of specific vitamin supplements (multivitamin, iron, vitamin D, vitamin B complex, and folic acid) were assessed via additional questionnaires. The Short Physical Performance Battery (SPPB) was performed at Wageningen University, in accordance with the protocol described by Guralnik et al. (23), with a 2.44-m gait speed, 5-times chair rise test, and a 3-position balance test.

### ProMuscle

A total of *n* = 122 participants were included in the ProMuscle trial (NCT01109628). Participants were recruited via a volunteer database, flyers, and information meetings. Participants were included when frail or prefrail according to the Fried criteria (24). Participants were excluded when they participated in resistance-type exercise programs in the 2 y before screening, or when they were diagnosed with any form of cancer, chronic obstructive pulmonary disease, diabetes, or renal insufficiency.

Dietary intake was assessed via 3-d food records on randomly assigned weekdays and weekend days. Food records were discussed with trained dietitians and household measures were used to optimize the portion size estimation. Nutrient intake was calculated using the Dutch food composition database of 2006. Dominant handgrip strength was measured by handheld dynamometry (Jamar, Jackson, MI) to the nearest 0.5 kg. Participants were seated on a chair without armrests, with their arms flexed at a 90-degree angle. The maximum effort of 3 attempts was used for analysis. The SPPB was performed similarly to the Nu-Age study, following the original protocol as described by Guralnik et al. (23).

### ProMO

The study population of ProMO (*n* *=* 81; NCT02683720) consisted of participants aged 65 y and older who were malnourished or at risk of malnutrition (a score <12 on the Mini Nutritional Assessment Tool—Short Form). Participants were recruited via dieticians, geriatric outpatient clinics of 2 hospitals (Rijnstate, Arnhem, Netherlands and Gelderse Vallei, Ede, Netherlands), the volunteer database of the university, and via advertisements in local and online media. Exclusion criteria were an expected life expectancy <12 mo, performing >2 h/wk of resistance exercise, planned increase in exercise during the study, impaired kidney function (estimated glomerular filtration rate <30 mL · min^−1^ · 1.73m^−2^, measured at baseline), lactose intolerance or milk protein allergy, use of corticosteroids (unless administered via inhaler or topically), use of diabetes medications, consumption of >19 portions of oral nutritional supplements in the previous month or >9 in the previous week, and consumption of >21 alcohol units per week.

Dietary intake was assessed by using 2-d food records on 2 consecutive days. Participants were interviewed by trained dietitians to optimize the completeness of the food records. Portion sizes were estimated by using household measures. The Dutch food consumption database 2011 was used to calculate the mean daily nutrient intake. Physical functioning was assessed by means of isokinetic hand grip strength and the SPPB. Participants performed the handgrip strength test 3 times per hand, while seated on a chair without armrests and with their elbows flexed at a 90-degree angle. The SPPB was assessed via the updated protocol of Guralnik et al. (25), with a balance test, 5-times chair rise test, and a 4-m gait speed test.

### V-Fit

Participants of the V-Fit trial (*n* *=* 207) were recruited via personal letters sent to senior residencies, home care organizations, general practitioners, and local advertisements. Eligible participants were aged 70 y and older, used care services, did not regularly exercise, and had a BMI ≤25 kg/m^2^ or experienced unintentional weight loss. Excluded were participants who were institutionalized, had a terminal disease, or were taking multivitamin supplements in the preceding month.

Dietary intake was assessed by a 3-d food record (2 weekdays and 1 weekend day). Participants were visited at home before and after the dietary intake assessment, to explain the procedure and to check completeness. The volumes of frequently used household measures were assessed to optimize portion size estimation. Nutrients were calculated by using the Dutch food consumption table of 1997. Physical functioning was measured by means of peak dominant hand grip strength and the Groningen Fitness test for Elderly. The latter is comparable to the SPPB, because it contains a balance test, 5-times chair rise test, and a 6-m gait speed test.

### Statistical methods

#### Graphical models

Graphical models are a marriage between probability theory and graph theory. They provide a powerful tool for dealing with uncertainty and complexity in statistics. Let $Z\, = \,( {{Z_{1,}}{Z_{2,}} \ldots ,{Z_p}} )\ $ denote a random vector with a joint distribution $p( Z )$. The conditional independence relations among random variables can be summarized in a graph $G\ = \ ( {V,\ E} )$, where *V* is a set of vertices (or nodes), and each vertex corresponds to a random variable, and *E* is a set of undirected edges. If 2 vertices ${Z_i},{Z_j} \in V$ form an undirected edge then we say that ${Z_i}$ and ${Z_j}\ $ are adjacent or connected and write $( {i,j} ) \in E$. Here, undirected means that $( {i,j} ) \in E$ is equivalent with $( {j,i} ) \in E$. Thus, undirected graphs represent symmetric relations. The absence of an edge between ${Z_i}$ and ${Z_j}$ corresponds with the conditional independence of the 2 random variables given the remaining variables under $p( Z )$ and is defined by:

(1) $$\begin{eqnarray*}
{Z_{i\ }}\amalg {Z_j}\ |\ {X_{V/ \left\{ {i,j} \right\}}}.\end{eqnarray*}$$

This is called the pairwise Markov property. Let ${X_{n\ \times p}}$ be the data matrix with *p* the number of variables in the network, and *n* the number of observations for each variable. In Gaussian graphical models, it is assumed that the vector of $X\ = ( {{X_{1,}}{X_{2,}} \ldots ,{X_p}} )\ $ follows a *p*-variate normal distribution ${N_p}( {\mu ,\ {{\boldsymbol{\Omega }}^{ - 1}}} )$ with mean $\mu $ and variance-covariance matrix $\Sigma \ = \ {{\boldsymbol{\Omega }}^{ - 1}}$, where ${\boldsymbol{\Omega }}$ represents the precision matrix. The partial correlation coefficients ${\rho _{ij|{\rm{rest}}}}$, which measure the correlations between ${X_i}$ and ${X_j}$ conditional on all other variables in the models, can be calculated as:

(2) $$\begin{equation*}
{\rho _{ij|{\rm{rest}}}} = - \frac{{{\omega _{ij}}}}{{\sqrt {\ {\omega _{ii}}} \ \sqrt {\ {\omega _{jj}}} }}\end{equation*}$$

where ${\rm{\ }}{\omega _{ij}}$, $i,j\ = \ 1,\ \ldots ,p$ are the elements of the precision matrix ${\boldsymbol{\Omega }}$.

#### CGMs

CGMs are a flexible type of graphical model, where we relax the multivariate normal distribution assumption for the joint distribution of $X\ = ( {{X_{1,}}{X_{2,}} \ldots ,{X_p}} )\ $. In fact, it can be applied to different types of data sets, such as non-Gaussian data, ordinal data, count data, and a mix of ordinal and continuous data. Mixed types of variable are common in food and nutrition data sets. In the proposed CGM we assume that there is an ordering for the possible values of each observed variable ${X_v},\ v\ V{X_v},\ v\ \in V$. This assumption holds if ${X_v}$ is binary, categorical with ordered categories, count, or continuous. We assume the dependence structures among the observed variables $X\ = ( {{X_{1,}}{X_{2,}} \ldots ,{X_p}} )$ are given by the Gaussian copula with a $p\ \times \ p$ correlation matrix. The Gaussian copula model can be constructed by introducing a vector of latent variables $Z\ = \ {Z}_{v} \,\, {N}_{p} ( 0,\ {\boldsymbol \Omega})$Ω that are related to the observed variables $X\ = \ {X_v}$ as:

(3) $$\begin{equation*}
{X_v} = \ F_v^{ - 1}\left( {\Phi \left( {{Z_v}} \right)} \right)
\end{equation*}$$

where ${F_v}$ denotes the marginal distribution of ${X_v}$ which are treated as nuisance parameters, and $\Phi $ is the cumulative distribution function of the standard normal distribution. As proposed by Behrouzi and Wit (18), we use CGMs to perform inference in the parameter ${\boldsymbol{\Omega }}$ of the Gaussian copula, which contains all the conditional independence relations between latent variables *Z*. The inference procedure of their approach is based on the penalized Expectation-Maximization (EM) algorithm, which iteratively computes the conditional expectation of the joint log-likelihood in the E-step, and optimizes this conditional expectation in the M-step. Furthermore, an ${\mathfrak{l}_1}$ regularization technique is used to put a grid of penalty parameters on the off-diagonal elements of ${\boldsymbol{\Omega }}$, which leads to a sparse matrix. The zeroes of ${\boldsymbol{\Omega }}$ correspond to the missing edges in the graph. A grid of regularization parameters $\Lambda \ = \ ( {{\lambda _1},\ \ldots ,\ {\lambda _N}} )$ determines the level of the sparsity of ${\boldsymbol{\Omega }}$. A different penalty results in a different graph structure. One approach to select an optimal graph is to compute various information criteria based on the observed penalized maximum log-likelihood. Because we are interested in graph estimation, we use the extended Bayesian information criterion (eBIC) (26) that has been introduced as follows:

(4) $$\begin{equation*}
{\rm{eBIC\ }}\left( \lambda \right) = - 2\, l\left( {{{{\boldsymbol{\hat{\Omega }}}}_\lambda }} \right) + \left\{ {\log \left( n \right) + 4\gamma \log \left( p \right)} \right\}d{f_\lambda },\end{equation*}$$

for conditional independence graph selection. Here, $d{f_\lambda }$ refers to the number of nonzero elements in the off-diagonal of ${{\boldsymbol{\hat{\Omega }}}_\lambda }$ and $\gamma \ \in [ {0,1} ]$ determines the strength of prior information on the size of the model space. We set $\gamma \ = \ 1/2$, because it was shown by Foygel and Drton (26) that for moderately large *n*, the eBIC with $\gamma \ = \ 1/2$ performs well to recover the underlying true graph. The optimal model has the minimum value of eBIC with respect to $\lambda $.

#### Inference uncertainty

In high-dimensional cases, there is often considerable uncertainty in the number of nonzero elements in the precision matrix. To compute uncertainty associated with the estimation of the precision matrix, we used a nonparametric bootstrap method to determine the statistical accuracy and the importance of each link in the estimated nutrients–physical functioning network. One can then choose only those links (or direct associations) that have a high probability of being present across all the bootstrap samples. For the penalized CGMs, we replicated *B* data sets that are generated by sampling with replacement from the data set ${X_{n \times p}}$. For each replicate, we ran the entire inference procedure of the CGMs, including the model selection to estimate an optimal graph. This nonparametric bootstrap reflects the underlying uncertainty in the estimated network. We implemented this procedure to calculate the uncertainty associated with the estimation of nutrients–physical functioning networks in the combined data.

#### Analysis

**FIGURE 1:**
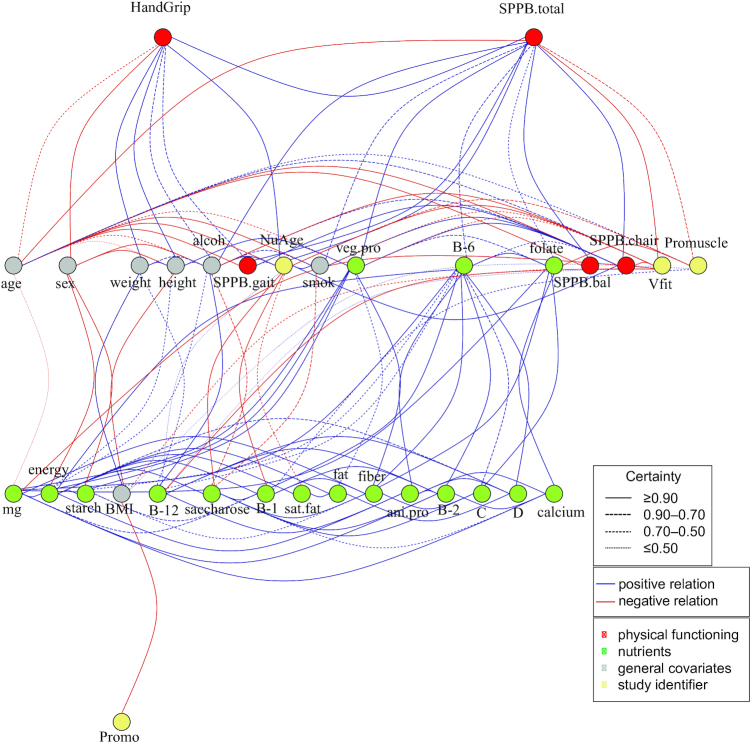
Nutrients-and-muscle-health networks for the combined data, showing simultaneous associations between nutrient intakes, measures of physical functioning, and the general covariates, which are inferred from copula graphical models. Edges represent conditional dependencies between nodes revealed by partial correlation coefficients. The absence of an edge between 2 nodes shows a conditional independence relation between them. The type of line used represents the certainty of the link, which is the frequency at which the conditional dependence relation (partial correlation) is found over 200 independent bootstraps. Alcoh, alcohol; ani.pro, animal protein; B-1, thiamin; B-2, riboflavin; B-6, vitamin B-6; B-12, vitamin B-12; bal, balance; C, vitamin C; D, vitamin D; mg, magnesium; sat.fat, saturated fat; smok, smoking; SPPB, short physical performance battery; veg.pro, vegetable protein.

The combined data set of the 4 studies contains *n* = 662 individuals and *P* = 33 variables. We grouped the variables into 3 categories: physical functioning, nutrient intakes, and general covariates. Different colors in the network represent variables and their corresponding categories (**Figure 1**, Results). We note that in the combined data set we kept the unit of all variables consistent across the various studies (e.g., energy intake is in kilojoules across all the 4 studies). Furthermore, we defined 4 dummy variables to represent each study to let the conditional independence property correct for associations that may arise from the different studies.

CGMs were used to construct the underlying connectivity networks for the combined data as well as for the individual studies. For each data set, we used our R package *nutriNetwork* to implement the CGM. More specifically, we used the *nutriNetwork* function with the default arguments. Furthermore, to select an optimal network across a grid of penalty parameters, we ran the *selectnet* function on the output object of the *nutriNetwork* function. The sparsity of the selected network is 0.23, where the sparsity level of the highest penalty term proposed by *nutriNetwork* is 0.05. To visualize networks we used the *plot* function in *nutriNetwork*.

Furthermore, we used a nonparametric bootstrapping approach to determine the uncertainty associated with the estimated links in the nutrients–physical functioning networks from the combined data. We generated 200 independent bootstrap samples from the combined data, as aforementioned. For each bootstrap sample we ran the entire inference procedure, including the model selection, using the *nutriNetwork* package. Furthermore, we calculated the frequency of each nonzero element of ${{\boldsymbol{\tilde{\Omega }}}^{( b )}}_{\hat{\lambda }}\ ( {b\ = \ 1,\ \ldots ,\ 200} )\ $ that was also nonzero in the estimated ${{\boldsymbol{\hat{\Omega }}}_{\hat{\lambda }}}\ $from the original combined data. In addition, we measured the fit of our model to the combined data. In **Supplemental Table 1** we show that the proposed CGM fits the data properly.

## Results

Baseline characteristics of all participants included in the analyses are presented in **Table 2**. Characteristics of the participants in the 4 trials were very comparable. Nonetheless, participants in the ProMO trial had a lower BMI than participants in the other 3 studies, and participants in the Nu-Age trial scored higher on the physical functioning tests (handgrip strength, total SPPB score, and chair rise test). The total sample consisted of more female than male participants, and participants scored relatively highly on the SPPB, with a mean score of 9.6 out of 12. Nutrient intake of the participants over the different trials is depicted in **Table 3**. Overall, the average intakes of nutrients were close to their RDAs for Dutch older adults (27). Only intake amounts of calcium [mean intake 992 mg/d, RDA 1200 mg/d (28)], folate [mean intake 243 µg/d, RDA 300 µg/d (29)], and vitamin D [mean intake 3.6 µg/d, RDA 20 µg/d (30)] were considerably lower than the RDA. However, the intake of nutrients via dietary supplements is not included in these intake amounts. The mean daily energy intake in the V-Fit study was lower than in the other trials, but the intake amounts of the nutrients were comparable across all studies.

**TABLE 2:** Baseline characteristics of included Dutch older participants^1^

Characteristics	Combined data (*n* = 662)	Nu-Age (*n* = 252)	ProMuscle (*n* = 122)	ProMO (*n* = 81)	V-Fit (*n* = 207)
Age, y	75 ± 7	71 ± 4	79 ± 8	74 ± 6	78 ± 6
Sex, % female	61.7	55.6	60.7	50.6	74.4
BMI, kg/m^2^	25.3 ± 3.8	26.1 ± 3.6	27.3 ± 4.2	22.0 ± 3.1	24.4 ± 2.7
Height, cm	167 ± 9	169 ± 8	166 ± 9	169 ± 9	164 ± 9
Weight, kg	71 ± 13	75 ± 13	75 ± 13	63 ± 11	66 ± 10
Alcohol intake, units/wk	9.4 ± 12.3	12.8 ± 11.9	10.1 ± 12.1	6.4 ± 5.1	5.4 ± 13.1
Smoker, % yes	30.9	49.6^2^	5.8	60.5^2^	10.8
Handgrip, kg	26 ± 9	29 ± 9	26 ± 9	21 ± 10	23 ± 8
SPPB total points (out of max 12)	9.6 ± 2.5	11.3 ± 1.1	8.0 ± 3.0	10.6 ± 2.1	8.1 ± 1.8
SPPB balance points (out of max 4)	3.5 ± 0.9	3.8 ± 0.6	3.1 ± 2.1	3.7 ± 0.8	3.5 ± 0.9
SPPB chair points (out of max 4)	2.6 ± 1.4	3.6 ± 0.7	1.9 ± 1.4	3.2 ± 1.1	1.3 ± 0.6
SPPB gait points (out of max 4)	3.5 ± 0.9	3.9 ± 0.4	3.0 ± 1.1	3.7 ± 0.6	3.2 ± 1.1

1 Values are mean ± SD unless stated otherwise. SPPB, Short Physical Performance Battery.

2 In ProMO and Nu-Age data, percentage smokers includes current and former smokers.

**TABLE 3:** Nutrient intake amounts of the Dutch older adults in the 4 substudies^1^

Characteristics	Combined data (*n* = 662)	Nu-Age (*n* = 252)	ProMuscle (*n* = 122)	ProMO (*n* = 81)	V-Fit (*n* = 207)
Energy, kcal/d	1913 ± 469	1908 ± 411	1964 ± 530	2177 ± 507	1779 ± 434
Animal protein, g/d	46 ± 15	46 ± 13	50 ± 18	50 ± 16	43 ± 14
Vegetable protein, g/d	28 ± 9	30 ± 8	26 ± 7	33 ± 12	23 ± 7
Fat, g/d	75 ± 24	73 ± 20	78 ± 25	91 ± 30	70 ± 22
Saturated fat, g/d	30 ± 11	27 ± 9	33 ± 11	37 ± 14	30 ± 10
Starch, g/d	105 ± 33	109 ± 31	100 ± 36	121 ± 40	96 ± 29
Sugar, g/d	106 ± 41	92 ± 32	122 ± 51	106 ± 36	114 ± 41
Fiber, g/d	22 ± 7	22 ± 6	22 ± 7	24 ± 9	21 ± 7
Thiamin, mg/d	1.0 ± 0.5	0.9 ± 0.3	1.2 ± 0.7	1.1 ± 0.5	1.1 ± 0.6
Riboflavin, mg/d	1.4 ± 0.5	1.4 ± 0.4	1.6 ± 0.6	1.6 ± 0.6	1.3 ± 0.5
Vitamin B-6, mg/d	1.5 ± 0.7	1.6 ± 0.5	1.6 ± 0.8	1.6 ± 0.8	1.4 ± 0.9
Folate, µg/d	243 ± 85	255 ± 71	189 ± 68	300 ± 115	239 ± 78
Vitamin B-12, µg/d	4.5 ± 2.7	5.0 ± 2.7	4.7 ± 2.9	5.0 ± 3.1	3.5 ± 2.2
Vitamin C, mg/d	104 ± 54	101 ± 48	113 ± 62	121 ± 68	95 ± 45
Vitamin D, µg/d	3.6 ± 2.3	3.6 ± 2.2	4.6 ± 3.1	3.6 ± 2.2	3.1 ± 1.7
Calcium, mg/d	992 ± 346	965 ± 309	1032 ± 394	1124 ± 386	946 ± 327
Magnesium, mg/d	318 ± 90	333 ± 77	329 ± 83	377 ± 104	267 ± 80

1 Values are mean ± SD.

Figure 1 represents the estimated network for the combined data. It shows interesting links between certain nutrients and functional outcome measures. Vitamin B-6 (pyridoxine), folate, and vegetable protein intake were positively and directly correlated with total SPPB score, with 88%, 53%, and 98% certainty based on bootstrap analysis, respectively. Vegetable protein and vitamin B-6 were also positively and partially correlated with chair rise test score (100% and 61% certainty, respectively), and this also held for vitamin B-12 (cobalamin, 50% certainty). These nutrients were not directly correlated with balance and gait scores, the 2 other parts of the total SPPB score. Based on the estimated network there was no direct association between handgrip strength and any of the nutrients. The proposed method shows that higher age and being female resulted in lower handgrip strength. Height, weight, and, surprisingly, alcohol intake were positively and directly correlated with handgrip strength. Besides having a lower handgrip strength, being female was also partially correlated with lower alcohol intake, lower energy intake, and lower starch intake. There was a negative partial correlation between intake of folate and BMI, whereas there was a positive partial correlation between intake of vitamin B-12 and body weight. In **Supplemental Figure 1**, we reported the corresponding relative frequencies of each link across the 200 bootstrap samples. In particular, in almost all bootstrap samples we inferred a direct link between vegetable protein and SPPB total (an index for measuring physical functioning). In other words, vegetable protein and SPPB total are partially correlated after adjusting for the effect of all remaining variables.

The ProMO study, which included malnourished participants, was negatively correlated with BMI after controlling for remaining variables. Intake of vegetable protein was positively and partially correlated with intakes of magnesium, starch, and fiber. Intake of folate was positively and partially correlated with intakes of fiber and vitamin C, and intake of calcium was partially correlated with intake of vitamin B-2 (riboflavin).

Visual overviews of the CGMs for the different substudies are presented in the supplementary data (**Supplemental Figures 2–5**), and corresponding partial correlation coefficients are presented in **Supplemental Figures 6–10**. **Table 4** provides an overview of the direct links between nutrients and measures of physical functioning that the models revealed in the combined data (letter A) and in the 4 different substudies (letters b–e). Note that in all data sets the model did not reveal links between intakes of animal protein, calcium, or magnesium and the functional outcome measures. Handgrip strength was partially correlated to intakes of energy, vegetable protein, thiamin, total fat, and saturated fat in substudies, but these links were not identified in the combined data. The other way around, intake of folate and vitamin B-12 intake were not linked to functional outcomes in the substudies, but were partially correlated with total SPPB score and chair rise test in the combined data.

**TABLE 4:** Overview of all direct links between nutrients and measures of physical functioning in Dutch older adults in the combined and separate trials^1^

	Energy	Animal protein	Vegetable protein	Total fat	Saturated fat	Fiber	Thiamin	Vitamin B-6	Folate	Vitamin B-12	Vitamin D	Calcium	Magnesium
Handgrip strength	c, e		d, e	c	c		c						
Total SPPB	c		A, c, e			c		A, d	A		d		
Balance			c										
Gait						c					d		
Chair			A			c		A, d		A			

^1^ *n* = 662. A, combined data; b, Nu-Age (no direct links); c, ProMuscle; d, ProMO; e, V-Fit.

### Nu-Age

The network of the Nu-Age (B, Supplemental Figure 2) study did not suggest any direct link between nutrients and functional outcome measures. In the Nu-Age network, the different SPPB components were only linked to each other, but not to any nutrient or general variable. This isolation can be explained by the average near-maximum scores on all SPPB components, in combination with the subsequent lack of variation. The absence of the expected links between SPPB components and handgrip strength underlines the profound isolation. Interestingly, age is not linked to handgrip strength. This is possibly caused by the relatively low mean (71 y) and maximum age (79 y) in the Nu-Age trial.

### ProMuscle

The network based on data of the ProMuscle (C, Supplemental Figure 3) study revealed interesting links between nutrients and function measures, which are often not observed in the other studies. Total fat and saturated fat intakes, as well as intake of thiamin and energy intake, were positively and partially correlated with handgrip strength. The data of ProMuscle revealed a positive partial correlation of fiber intake with chair rise test, gait speed, and total SPPB scores. The network based on ProMuscle data was the only network that revealed a link between a nutrient and balance score. There was a positive partial correlation between vegetable protein and this balance score, as well as with total SPPB score after removing the effect of remaining variables. Total energy intake was partially correlated with total SPPB score, and this was also identified by the estimated physical functioning networks based on the combined data.

### ProMO

The network of the ProMO (D, Supplemental Figure 4) study showed a positive partial correlation between vegetable protein intake and handgrip strength. Moreover, vitamin B-6 intake was shown to be positively related to chair rise test score and total SPPB score, which was also identified by the network of the combined studies. The network based on ProMO data was the only network that identified direct links between vitamin D and physical functioning. Vitamin D was positively correlated with gait speed and total SPPB score, after removing the effect of remaining variables.

### V-Fit

Also in the network of the V-Fit (E, Supplemental Figure 5) study, positive correlations of vegetable protein intake with handgrip strength and with total SPPB score were observed. Here, total energy intake was also positively related to handgrip strength. The other nutrients in the V-Fit study were not partially correlated with physical functioning measures.

## Discussion

The field of nutritional epidemiology faces mounting criticism over the methodological approaches it uses. In nutritional epidemiology, associations are mainly assessed via conventional methods like univariate regression (31–33) and principal components analysis (34–36). Associations between nutrient intake and health outcomes are prone to confounding by other variables, and intake amounts of different nutrients are often partially correlated with each other (37). CGMs, which are frequently used in various research areas like genomics and metabolomics, can deal with these common caveats in the field of nutritional epidemiology.

A main advantage of the CGM is its ability to distinguish between direct and indirect associations among dietary intake variables. This is due to the estimation of a precision matrix (the inverse of the variance-covariance matrix) that contains all conditional (in)dependence relations among the variables. Conventional methods such as principal components analysis use marginal correlations that do not adjust for the indirect effect of other variables when assessing pairwise associations. Removing indirect effects is crucial in association studies, because it advances our understanding of the underlying mechanisms that generated the data. Sparse structure learning is another advantage of this study which facilitates interpretation of results. The proposed method is an exploratory tool that can be applied to dietary intake non-Gaussian data and mixed ordinal/continuous data to uncover conditional independence networks between dietary variables. Also, the method can deal with missing values, which are common in nutritional epidemiology, due to the employed EM algorithm. In addition, the conventional methods only adjust for preselected confounding variables (38), whereas the proposed CGM avoids the preselection step. In fact, it handles high-dimensional data, where the number of variables can exceed the number of samples. As a result, it addresses confounding by using conditional dependence relations (or *partial correlations*), where measured associations are adjusted for the influence of all remaining variables in the data set. The major advantage of the proposed CGM over conventional methods is its ability to distinguish between direct associations (conditional dependence relations) and indirect associations (marginal dependencies or Pearson correlations). Thus, applying the proposed CGM in nutritional research is appealing, because it addresses the main caveats in nutritional epidemiology by detecting direct associations.

In this study, we used the proposed CGM to detect simultaneous associations between nutrients and measures of physical functioning. Via this method, we detected interesting links between nutrient intake and physical functioning. First, the estimated network of the combined studies shows that intake of vegetable protein plays a role in physical functioning, because it was related to improved total SPPB score and chair rise test score. Interestingly, we did not observe any direct links between animal protein intake and physical functioning, although animal protein is often considered a better source to promote physical functioning than vegetable protein.

Longitudinal studies have found that high animal protein intake attenuates strength loss over 6 y of follow-up (39), and, if combined with physical activity, decreases the risk of developing disabilities (40). Based on current knowledge, the consensus is that animal protein has a higher potency in stimulating muscle protein synthesis than vegetable protein (41). This superiority of animal protein over vegetable protein is characterized by a number of studies comparing animal protein sources with soy protein or wheat protein (42–47). In a recent review by Gorissen et al. (48), the authors emphasize the large differences in amino acid profiles of various plant sources. They point out that specific plant-based proteins (for instance potato protein) have an amino acid composition that is much more similar to that of animal protein than the amino acid profile of wheat and soy protein. Our data are based on food intake of exclusively Dutch older adults (49). Food consumption research in this population shows that <1% of their vegetable protein intake is soy-based. Although wheat protein is, with ∼50%, the largest source of vegetable protein intake in this population, there are other protein sources with a notable contribution to vegetable protein intake (vegetables, 9%; potatoes, 7%; fruits and nuts, 7%). Therefore, it is possible that the suspected superiority of animal protein over vegetable protein does not fully apply to this data set of an almost non–soy consuming population.

Besides, it is even possible that vegetable protein has beneficial effects over animal protein on muscle health. The alkaline properties of vegetable protein–containing foods are hypothesized to prevent the muscle wasting response to acidosis, thereby conserving muscle mass during ageing (50). Acute supplementation with the alkaline bicarbonate resulted in a modestly improved lower extremity strength in older women, but not in men (51). Sahni et al. (51) showed that animal protein was positively associated with leg lean mass, but vegetable protein was associated with increased quadriceps strength. The authors suggested that vegetable protein might be linked to improved muscle strength (independent of muscle mass), or that vegetable intake behaves as a marker of overall dietary quality. This latter explanation is strengthened by their finding that adjustment for fruit and vegetable intake attenuates the association between vegetable protein intake and quadriceps strength. In our study, vegetable protein intake was related to the chair rise test, a test which relies primarily on quadriceps strength (52). Although our networks did not include adjustments for fruit and vegetable intake, we did adjust for dietary fiber and a broad range of other nutrients, which removes large amounts of dietary quality confounding. Still, overall dietary quality could confound this association, because the model did not include data on intake of other compounds in the food matrix of vegetable protein–containing products.

All in all, the positive and direct association of vegetable protein (and not animal protein) with physical functioning in this study can be explained via *1*) a relatively high ingestion of higher-quality vegetable protein in this Dutch population, *2*) muscle mass–conserving properties of the alkaline properties of vegetable protein–containing foods, and *3*) other, yet to be unraveled mechanisms via which vegetable protein may improve muscle quality.

Second, and similarly to intake of vegetable protein, intake of vitamin B-6 was also directly associated with total SPPB score and chair rise test score. Two other B-vitamins, folate and vitamin B-12, were linked to total SPPB score and chair rise test score, respectively. The exact roles of these B-vitamins in physical functioning are unclear. However, we know that vitamin B-6, vitamin B-12, and folate act together in the one-carbon pathway, via which they lower homocysteine concentrations. Homocysteine concentrations have been shown to be negatively associated with quadriceps strength (53), chair rise test (54), and gait speed (53–55). A few studies suggest that elevated homocysteine concentrations can hamper physical functioning by inducing mitochondrial dysfunction (56–58). Besides, elevated homocysteine concentrations cause endothelial dysfunction via increased oxidative stress (59), and are associated with increased white matter hyperintensities, which especially affects lower extremity functioning (60, 61). This is in line with our findings, where no association was found with handgrip strength, but only with functional outcomes related to the lower extremities.

Previous studies assessing the relation between the intake of these B-vitamins and physical functioning show conflicting results. Intake amounts of vitamin B-6, but not folate and vitamin B-12, were associated with improved mobility (62). Alternatively, intakes of vitamin B-6 and folate, but not vitamin B-12, were associated with a lower frailty risk in older adults (63), and intake amounts of vitamin B-6 and folate, but not vitamin B-12, were lower in sarcopenic than in nonsarcopenic older adults (64). Considering the clear associations of homocysteine concentrations and physical functioning, and the role of intake of vitamin B-6, folate, and vitamin B-12 in one-carbon metabolism, it is likely that adequate intake of these vitamins helps in maintaining physical functioning. The different findings over the studies investigating the association between B-vitamin intake and physical functioning could mean that over the different populations different B-vitamins were limiting in one-carbon metabolism. Otherwise, it could mean that these individual B-vitamins act on additional, yet to be identified mechanisms in relation to physical functioning.

Besides the use of the CGMs, this study has another major strength: the assessment of dietary intake data via multiple-day food records. This is considered an accurate way to collect data on intake of multiple nutrients, performing better than other frequently used methods such as 24-h recalls or FFQs (65). A drawback of this study is that data on some confounders were not available. In the design phase of the substudies, decisions were made on which outcomes to measure. These decisions can be viewed as a form of a priori confounder identification that is still present within our approach. Most importantly, we did not have data on physical activity, which can be a major confounder in the intake–function relation. We did however partly overcome this issue by correcting for total energy intake and BMI, which together roughly resemble physical activity level.

There are some potential limitations of the proposed CGM. First, nonordinal categorical variables such as occupation types, race, and religions cannot be taken into account explicitly. However, it is possible to include them in the model via dummy variables. Second, estimation of graph structure is a challenging problem in graphical models where a range of tuning parameters control the sparsity level of a graph. Different methods (e.g., extended Bayesian/Akaike information criteria, cross-validation, and the stability selection approach) have been proposed for structure learning in graphical models. However, different methods may differ by having a few additional or fewer links. Thus, it is always sensible to measure the underlying uncertainty of an estimated parameter. Third, Gaussian CGMs often employ 1 parameter to model the interaction between a pair of variables and no parameters are available for higher-order interactions between ≥3 variables. This is computationally convenient, clearly, but this may not correspond to reality. In fact, it is always sensible to do post hoc checks to see if the fitted model provides a satisfactory fit to the data.

In conclusion, Gaussian CGMs are a powerful exploratory method that can be used to reconstruct dietary intake networks from high-dimensional data. They reveal partial correlations between dietary variables which may help us to identify important nutritional factors in age-related declines in physical functioning. In this study we found that greater vegetable protein, folate, and vitamin B-6 and B-12 intakes were partially correlated with improved functional outcome measurements in Dutch older adults.

## Supplementary Material

nxz269_Supplemental_Files

## Notes

Supported by Top consortium for Knowledge and Innovation Agri and Food, Wageningen, Netherlands.

Author disclosures: The authors report no conflicts of interest.

Supplemental Table 1 and Supplemental Figures 1–10 are available from the “Supplementary data” link in the online posting of the article and from the same link in the online table of contents at https://academic.oup.com/jn/.

PB and PG contributed equally to this work.

Abbreviations used: CGM, copula graphical model; eBIC, extended Bayesian information criterion; EM, Expectation-Maximization; SPPB, Short Physical Performance Battery.
