# Supplementary material for: Dietary Intakes of Vegetable Protein, Folate, and Vitamins B-6 and B-12 Are Partially Correlated with Physical Functioning of Dutch Older Adults Using Copula Graphical Models
Source: J Nutr. 2019 Dec 20;150(3):634–43. doi: 10.1093/jn/nxz269 (PMC7056616; doi:10.1093/jn/nxz269)
Supplement: nxz269_Supplemental_Files [file nxz269_supplemental_files.zip › Supplemental figure10_page10.pdf]

Dietary intakes of vegetable protein, folate, and vitamins B-6 and B-12 are partially correlated with improved physical functioning of Dutch older adults using Copula Graphical Models. Pariya Behrouzi, Pol Grootswagers

Online Supplementary Material

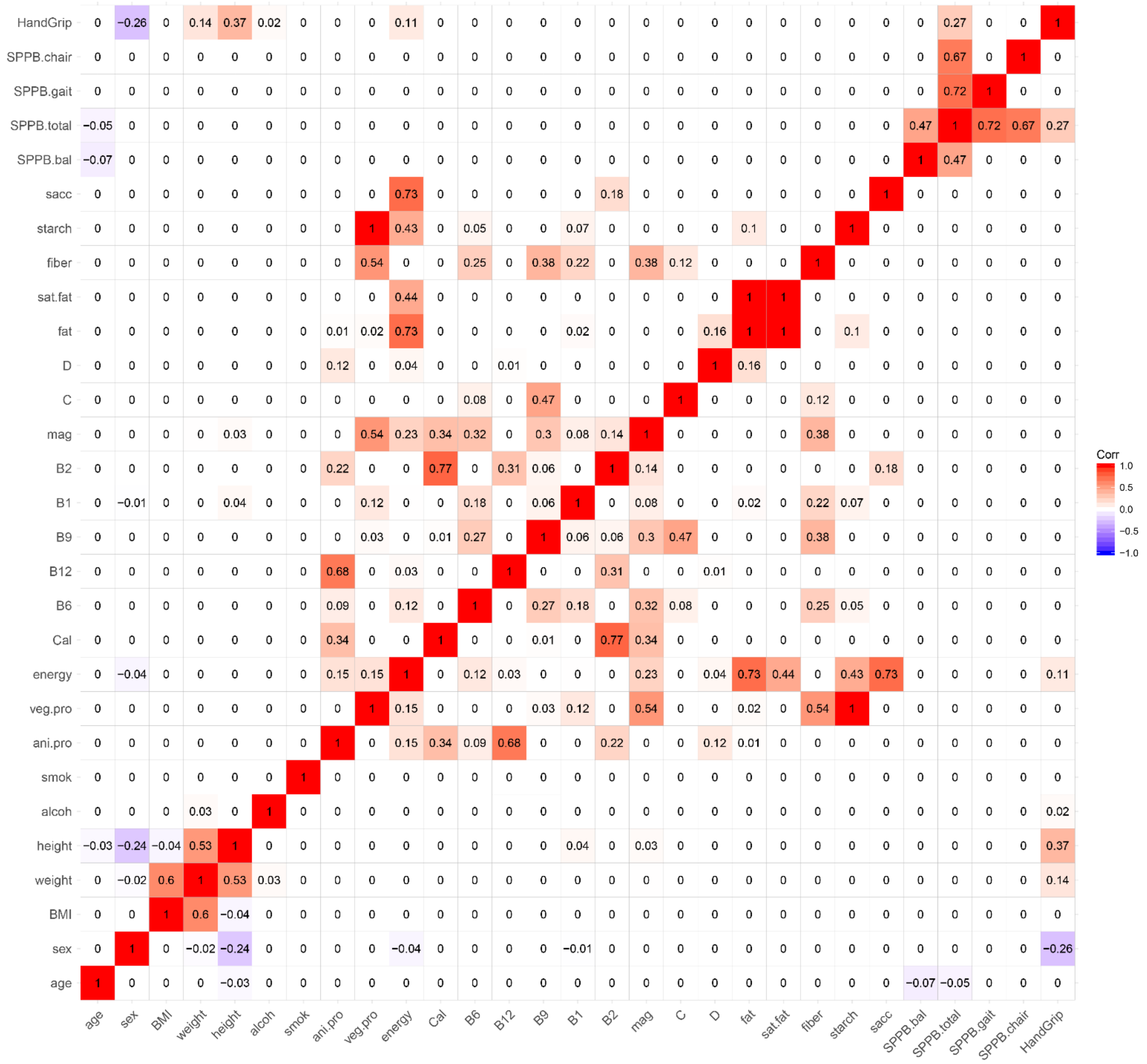

Supplemental Figure 10 The partial correlation coefficients associated with each link in the estimated network for the V-Fit study (*n*=207). Each element represents the partial correlation coefficient between two variables.
