# Supplementary material for: Dietary Intakes of Vegetable Protein, Folate, and Vitamins B-6 and B-12 Are Partially Correlated with Physical Functioning of Dutch Older Adults Using Copula Graphical Models
Source: J Nutr. 2019 Dec 20;150(3):634–43. doi: 10.1093/jn/nxz269 (PMC7056616; doi:10.1093/jn/nxz269)
Supplement: nxz269_Supplemental_Files [file nxz269_supplemental_files.zip › Supplemental figure1_page1.pdf]

Dietary intakes of vegetable protein, folate, and vitamins B-6 and B-12 are partially correlated with improved physical functioning of Dutch older adults using Copula Graphical Models. Pariya Behrouzi, Pol Grootswagers

Online Supplementary Material

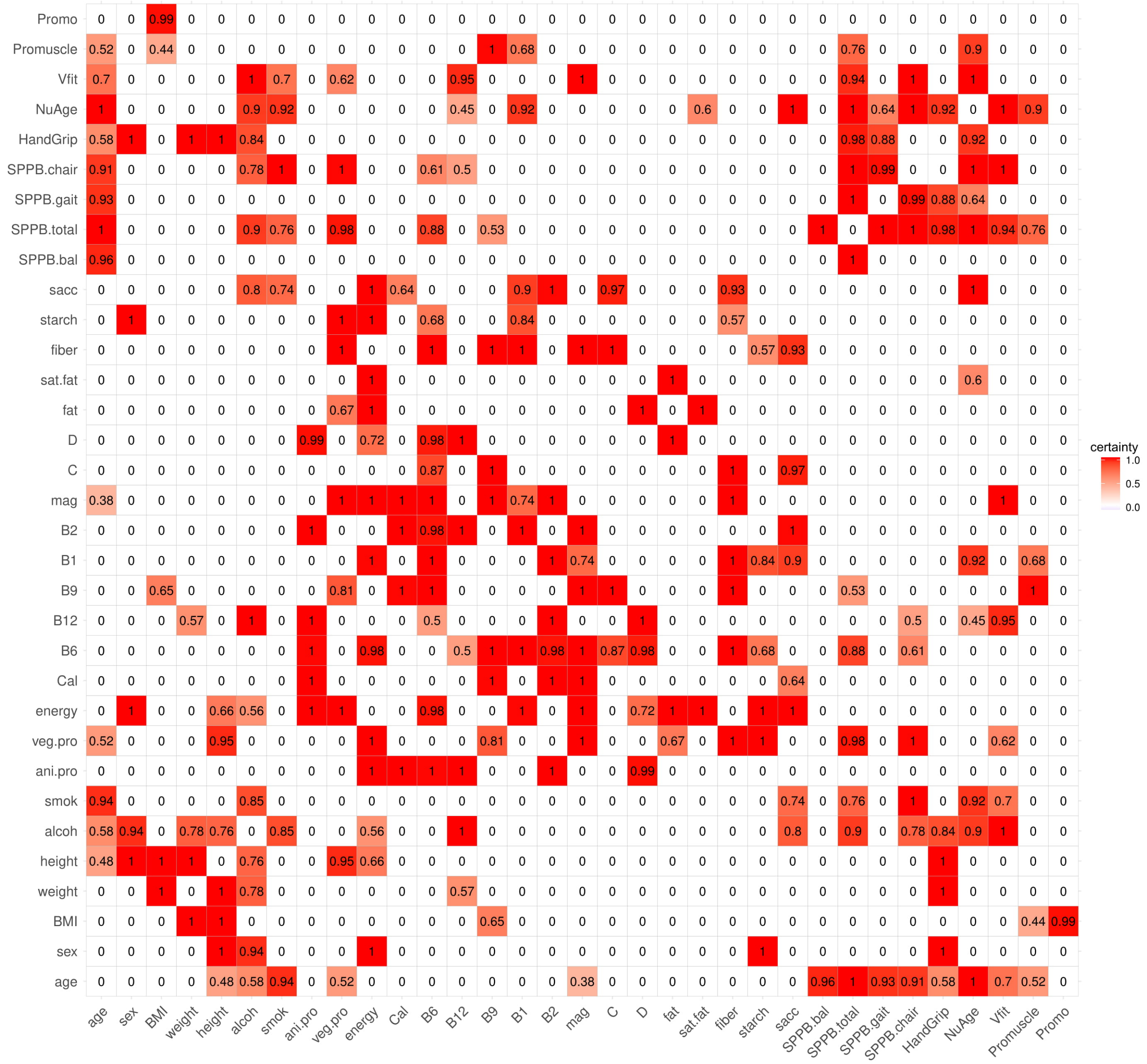

Supplemental Figure 1 The uncertainty associated with each link in the estimated network for the combined data (*n*=662) using a non-parametric bootstrap. Each element represents the relative frequency of having link between variables in bootstrap version of the combined data. Here, we generated 200 independent bootstrap samples. For instance, direct link between vegetable protein (veg.pro) and SPPB total was presented in 98% of all bootstrap samples, and link between vegetable protein and B9 was presented in 81% of all bootstrap samples.
