# Supplementary material for: Dietary Intakes of Vegetable Protein, Folate, and Vitamins B-6 and B-12 Are Partially Correlated with Physical Functioning of Dutch Older Adults Using Copula Graphical Models
Source: J Nutr. 2019 Dec 20;150(3):634–43. doi: 10.1093/jn/nxz269 (PMC7056616; doi:10.1093/jn/nxz269)
Supplement: nxz269_Supplemental_Files [file nxz269_supplemental_files.zip › Supplemental figure5_page5.pdf]

Dietary intakes of vegetable protein, folate, and vitamins B-6 and B-12 are partially correlated with improved physical functioning of Dutch older adults using Copula Graphical Models. Pariya Behrouzi, Pol Grootswagers

Online Supplementary Material

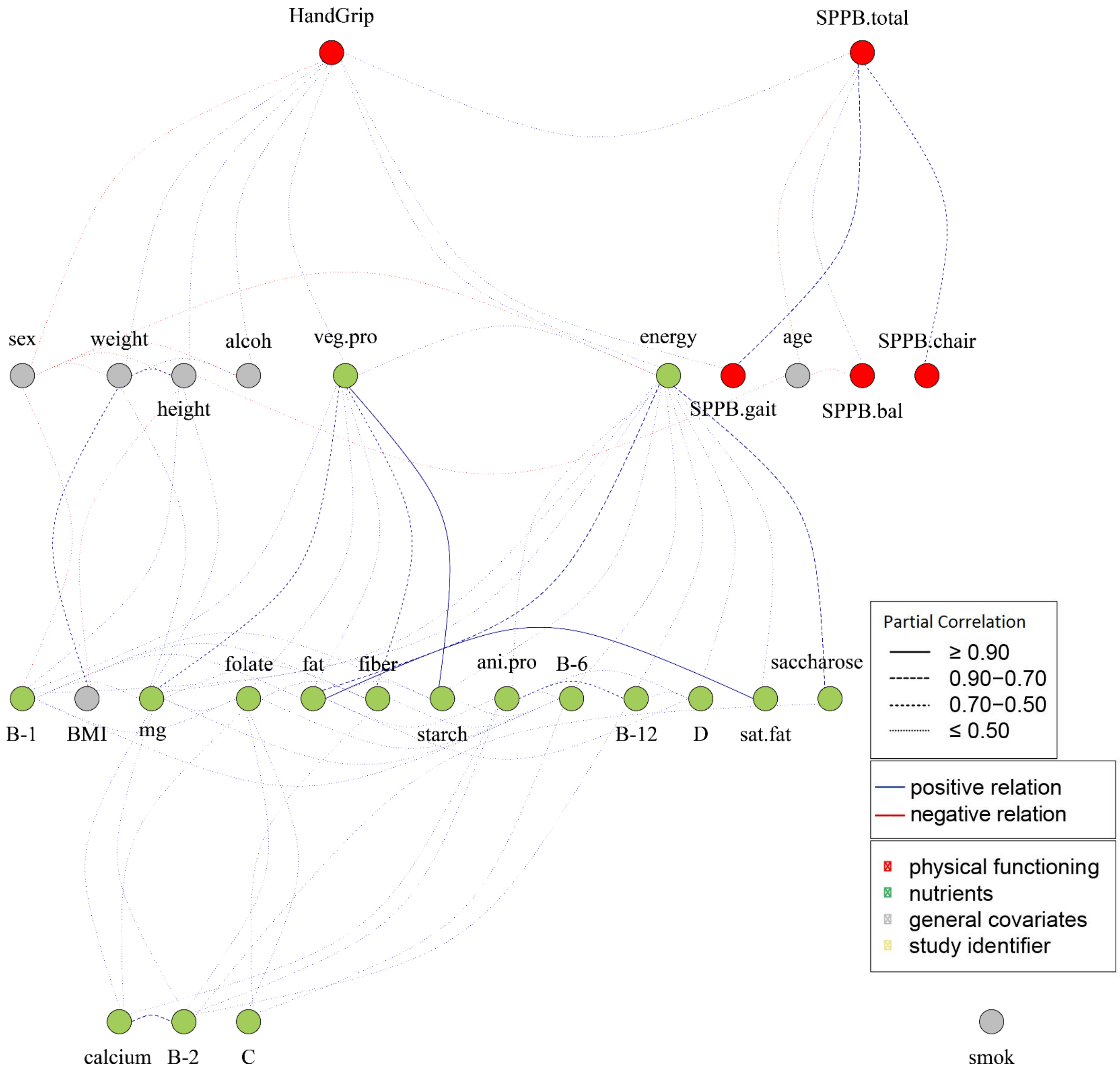

Supplemental Figure 5 Conditional dependencies networks among variables in the V-Fit study (n=207).

The type of line used represents the strength of each edge based on partial correlation values. This study, individually, suggests that vegetable protein is directly associated to the level of physical functioning. And most of the other nutrients influence the physical functioning items via vegetable protein and energy.
