# Supplementary material for: Dietary Intakes of Vegetable Protein, Folate, and Vitamins B-6 and B-12 Are Partially Correlated with Physical Functioning of Dutch Older Adults Using Copula Graphical Models
Source: J Nutr. 2019 Dec 20;150(3):634–43. doi: 10.1093/jn/nxz269 (PMC7056616; doi:10.1093/jn/nxz269)
Supplement: nxz269_Supplemental_Files [file nxz269_supplemental_files.zip › Supplemental figure6_page6.pdf]

Dietary intakes of vegetable protein, folate, and vitamins B-6 and B-12 are partially correlated with improved physical functioning of Dutch older adults using Copula Graphical Models. Pariya Behrouzi, Pol Grootswagers

Online Supplementary Material

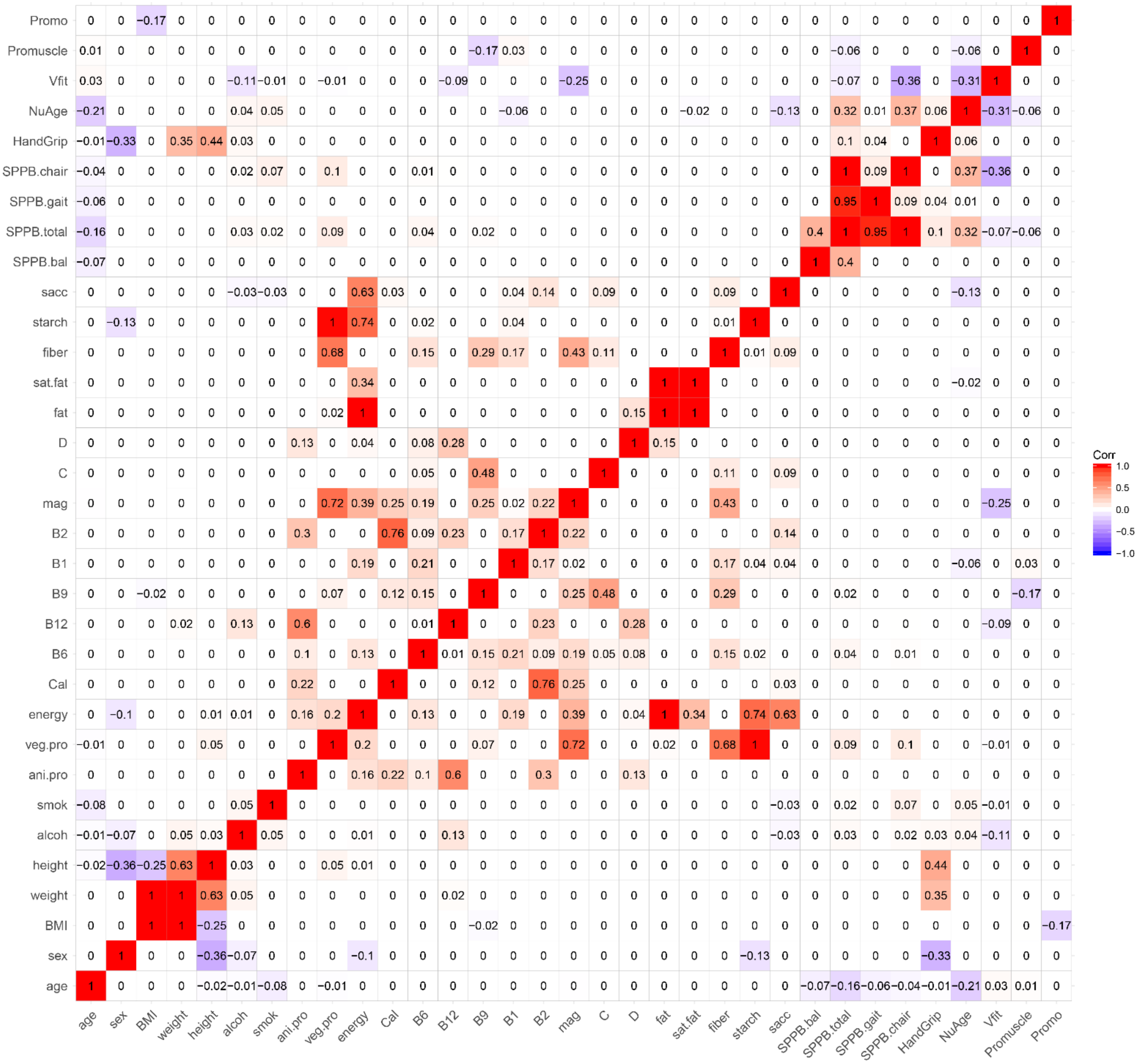

Supplemental Figure 6 The partial correlation coefficients associated with each link in the estimated network for the combined data (*n*=662). Each element represents the partial correlation coefficient between two variables.
