# Supplementary material for: Dietary Intakes of Vegetable Protein, Folate, and Vitamins B-6 and B-12 Are Partially Correlated with Physical Functioning of Dutch Older Adults Using Copula Graphical Models
Source: J Nutr. 2019 Dec 20;150(3):634–43. doi: 10.1093/jn/nxz269 (PMC7056616; doi:10.1093/jn/nxz269)
Supplement: nxz269_Supplemental_Files [file nxz269_supplemental_files.zip › SupplementalTable1_page11.pdf]

Fit of model to integrated data

One way to assess the adequacy of the proposed model is to compare the fitted model with the saturated model. Let $l_m(\hat{\boldsymbol{\Omega}})$ and $l_s(\hat{\boldsymbol{\Omega}})$ be the log-likelihood of the observations for the fitted model and the saturated model, respectively. The deviance statistics which is defined as

$$D = l_m(\hat{\boldsymbol{\Omega}}) - l_s(\hat{\boldsymbol{\Omega}})$$

allows us to assess the goodness-of-fit of the proposed method. Table 1 shows how well the proposed model fits the data. The chi-square test with 401 degrees of freedom gives a p-value of 1, indicating that the proposed model fits the data adequately**.**

Supplemental Table 1 A summary of model fit to the integrated data

Model	df	Log-likelihood	Deviance	P-value
Fitted model	128	−4847.58		
Saturated model	528	−1856.94		
Fitted model vs Saturated model	401		5981.28	1
